# What did the pandemic teach us about effective health communication? Unpacking the COVID-19 infodemic

**DOI:** 10.1186/s12889-022-14707-3

**Published:** 2022-12-13

**Authors:** Eric J. Cooks, Melissa J. Vilaro, Brenda W. Dyal, Shu Wang, Gillian Mertens, Aantaki Raisa, Bumsoo Kim, Gemme Campbell-Salome, Diana J. Wilkie, Folake Odedina, Versie Johnson-Mallard, Yingwei Yao, Janice L. Krieger

**Affiliations:** 1 STEM Translational Communication Center, College of Journalism and Communications, University of Florida, Weimer Hall 2043, PO Box 118400, Gainesville, FL 32611-8400 USA; 2 Department of Family, Youth, and Community Sciences, University of Florida, Gainesville, USA; 3 Department of Biobehavioral Nursing Science, University of Florida, Gainesville, USA; 4 Department of Biostatistics, University of Florida, Gainesville, USA; 5 Department of Media and Communication, Joongbu University, Geumsan, South Korea; 6 Department of Population Health Sciences, Geisinger, Danville, USA; 7 Department of Quantitative Health Sciences, Mayo Clinic, Jacksonville, USA; 8 College of Nursing, Kent State University, Kent, USA

**Keywords:** COVID-19, Health information-seeking, Health communication, Communication sources, Evidence-based campaigns

## Abstract

**Background:**

The spread of unvetted scientific information about COVID-19 presents a significant challenge to public health, adding to the urgency for increased understanding of COVID-19 information-seeking preferences that will allow for the delivery of evidence-based health communication. This study examined factors associated with COVID-19 information-seeking behavior.

**Methods:**

An online survey was conducted with US adults (*N* = 1800) to identify key interpersonal (e.g., friends, health care providers) and mediated (e.g., TV, social media) sources of COVID-19 information. Logistic regression models were fitted to explore correlates of information-seeking.

**Results:**

Study findings show that the first sought and most trusted sources of COVID-19 information had different relationships with sociodemographic characteristics, perceived discrimination, and self-efficacy. Older adults had greater odds of seeking information from print materials (e.g., newspapers and magazines) and TV first. Participants with less educational attainment and greater self-efficacy preferred interpersonal sources first, with notably less preference for mass media compared to health care providers. Those with more experiences with discrimination were more likely to seek information from friends, relatives, and co-workers. Additionally, greater self-efficacy was related to increased trust in interpersonal sources.

**Conclusion:**

Study results have implications for tailoring health communication strategies to reach specific subgroups, including those more vulnerable to severe illness from COVID-19. A set of recommendations are provided to assist in campaign development.

**Supplementary Information:**

The online version contains supplementary material available at 10.1186/s12889-022-14707-3.

Since first identified in December 2019, the novel SARS-CoV-2 (COVID-19) virus has left a trail of death and economic disruption in its wake. In the United States (US) alone, the COVID-19 pandemic has caused more than 1 million deaths, with many more likely due to reporting errors [1, 2]. The spread of COVID-19 can be mitigated through strategies such as mask-wearing and social distancing in public settings, and while the development of vaccines and therapeutics offer effective options for prevention and care, hesitancy and non-compliance with these treatments and strategies remains a considerable problem [3]. While new and more contagious variants continue to emerge, we have also witnessed the spread of conspiracy theories on virus origin, racist threats, and suspicion towards public health institutions that present significant challenges to public health measures [4–6].

This rise in COVID-related incivility, skepticism, and conspiracy beliefs can partly be attributed to high levels of dis/misinformation and distrust in media that has been described as a “hidden epidemic” [7, 8]. As efforts to control virus spread in the US reduced opportunities for face-to-face communication, individuals turned instead to social media, TV news, and other mass media platforms often littered with inaccuracies in search of COVID-19 information. As this information went viral and became widely spread many of these “fake news” stories became ubiquitous in American culture. The spread of unvetted scientific information presents a significant challenge to public health efforts, adding to the urgency for increased understanding of information-seeking preferences during the COVID-19 pandemic that will ultimately allow for the tailored delivery of evidence-based health communication through these preferred sources and channels.

## Information-seeking

According to the Protective Action Decision Model (PADM) [9], behavioral response to health risks depends partly on information exposure. By receiving timely and accurate information about COVID-19, individuals are better equipped to formulate accurate risk perceptions and engage in preventive steps [10–13]. Following this logic, it is essential that evidence-based COVID-19 information be translated in a manner that meets the needs of diverse stakeholder groups by understanding the factors associated with health information-seeking behavior (HISB). One strategy for understanding HISBs during the pandemic is to explore preferences for the first sought information source as an indicator of persuasiveness [14], and for sources deemed most trustworthy as a proxy for credibility [15].

The concept of uncertainty is important to HISB. The novelty of COVID-19 and lack of societal preparedness increased uncertainty in how to respond [16], increasing the likelihood that individuals will seek to manage this uncertainty by searching for relevant information [17–19]. Related to this idea of HISB as a tool to manage uncertainty is self-efficacy, which refers to the extent to which one believes in their ability to successfully perform a behavior [20]. Prior to engaging in a HISB, individuals have a tendency to first develop outcome expectations and evaluate whether they possess the ability to enact this search [21].

HISB during the pandemic operates within the context of advances in mass media technology, with increased use of digital media platforms (e.g., Internet search engines, social media) and the associated concerns regarding false information [22, 23]. In addition to information received from interpersonal sources (e.g., friends, family, health care providers), information consumers now have diverse opportunities to seek and obtain health-related information with platforms such as Facebook, YouTube, and Google providing 24/7 access to information of varying quality [12]. The affordances of these platforms (e.g., sharing, liking, commenting) allow for an enhanced ability to create, receive, and disseminate health information. This increased media choice also allows for selective exposure to like-minded voices, which can lead to increased perceptions of bias within the general media [24]. Further, media slant towards a specific political ideology or issue position can be extreme within these mediated settings, with exposure having an influence on COVID-19 incidence [25]. All told, preferences for health information in the current media landscape warrant exploration to assess how audience factors are related to HISB.

While technological advancements have placed a wealth of information at our fingertips, there are disparities in who utilizes and benefits from these technologies based in part on longstanding social and digital inequalities [26, 27]. While it has been argued that groups often marginalized by society (e.g., inequality based on age, gender, educational attainment, etc.) are simply lagging behind the curve in uptake of these technologies and will eventually bridge the gap, many of these groups often require government intervention to stimulate use and are more likely to discontinue use once begun [28]. Also, for these marginalized groups, self-efficacy in the use of technology is likely to be lower compared to those with more capital [29]. Therefore, while mass media technology provides wide reach and convenience to many, the associated inequities in use suggest that health communication campaigns seeking to tailor dissemination strategies should attend to audience features that may point to source preferences.

The COVID-19 pandemic illuminated how racial and ethnic discrimination can be amplified via the media, making HISB difficult for some groups. Anti-Asian sentiment, fueled in part by social media, has seen a dramatic increase during the pandemic, and politicians have used this crisis to propagate stereotyping and discriminatory policies against racial and ethnic minorities [30–32]. Black Americans who have historically been confronted with significant racism and discrimination in the US also report that their experiences with discrimination have increased during COVID-19 [33]. This lived discrimination can act as a biological stressor for which individuals must develop coping strategies, such as information-seeking [34, 35].

## Study aim

Given that uncertainty about COVID-19 has increased HISB [36, 37], focused effort is needed to deliver evidence-based health information through preferred sources in order to combat mis/disinformation and improve population health. Prior work has demonstrated that self-efficacy is positively associated with the frequency of HISB during the pandemic [38, 39]. However additional work is needed to explore the sources people seek out first and which ones they trust the most, particularly in relation to confidence in information-seeking ability. In some cases, first sought and most trusted sources may be the same. In other cases, the sources that are most readily available to an individual may not be the most trusted. For example, some individuals may find health care providers to be highly trustworthy, but they are unavailable 24/7 to meet information needs.

Digital inequalities have also likely been exacerbated during the pandemic as marginalized groups are unable to offset the loss of in-person communication [40]; these factors may contribute to differences in HISB [41]. Further, increases in perceived discrimination may be associated with information-seeking strategies during the pandemic [42]. Building on previous work related to HISB during COVID-19 [41, 43], the aim of this study was to investigate individual preferences for the first sought out and most trusted sources of COVID-19 information to guide tailored campaign development.

### Research questions

- RQ1: Are sociodemographic characteristics associated with preferences for (a) first sought and (b) most trusted source of COVID-19 information?
- RQ2: Are discrimination and self-efficacy associated with preferences for (a) first sought and (b) most trusted source of COVID-19 information?

## Methods

### Study design and participant recruitment

Using a cross-sectional study design, between September and November 2020, a period that saw approximately 94,000 deaths from COVID-19 in the US (Johns Hopkins COVID-19 Tracker https://coronavirus.jhu.edu/us-map), US adults aged ≥ 18 years (*N* = 1800) recruited through a panel owned by a cloud-based survey platform completed the online Florida Health Ancestry Study survey (FHAS). The sampling framework was specified so that quotas would represent the general US adult population (see Table 1).

Participants meeting these inclusion criteria received an electronic link to the survey. Partial responses were not recorded, but all participants were given one week to complete the survey. The “Forced Response” validation was used for all items, although participants could select “prefer not to answer.“ A $15.00 incentive was mailed to participants who completed the survey. The University of Florida Institutional Review Board (IRB201901264) approved this study with a waiver of documentation of informed consent.

**Table 1:** Sampling framework

Variable	National Sample (US general population)		
**Age**	18–24	12.8%	
	25–34	17.7%	
	35–44	16.7%	
	45–54	17.7%	
	55–64	16.4%	
	65+	18.8%	
**Gender**			
Female	50.8%		
Male	49.2%		
**Race**			
Non-Hispanic White	61.9%		
Non-Hispanic Black	12.3%		
Hispanic	17.4%		
Asian	5.3%		
American Indian/Alaskan Native	0.7%		
Other Race	2.5%		

### Instrument

Participants completed the 48-item FHAS survey developed using the behavioral core measures from NCI-designated cancer center catchment area supplements [44]. The FHAS includes investigator-derived measures related to COVID-19, perceived discrimination, and self-efficacy in obtaining health information (see supplement “additional_file_1” for more information on items used in this analysis). For all items, responses of “Don’t know” and “Prefer not to answer” were treated as missing.

### Measures

#### COVID-19 information-seeking

To measure the first sought and most trusted sources of information about COVID-19, participants responded to two items (the COVID-19 questions in this study were adapted from a Palliative Care & Supportive Oncology Workgroup Survey and the eHealth Literacy Scale [45]), (“When you had a strong need to get information about COVID-19, where did you FIRST go to get information?“; “When you had a strong need to get information about COVID-19, which of the following did you find to be the MOST trusted as a source of information about coronavirus or COVID-19?“). For the univariable and multivariable analyses, response options for both items were dichotomized into the following sources: “Mass media” (Internet: Google or another search engine/WebMD or another medical website; Printed materials: newspapers, magazines; Social media: Facebook, Instagram, Twitter; Television) and “Interpersonal” (Conversations with people you trust: friends, relatives, or co-workers; Health care provider: doctor, nurse, social worker). Responses of “Other (Please specify:)” were treated as missing.

#### Self-efficacy

On a 5-point scale where 1 = “Not confident at all” and 5 = “Completely confident,“ self-efficacy was measured as confidence in obtaining general health information using a single item [46], “Overall, how confident are you that you could get advice or information about health or medical topics if you needed it?“ (M = 4.1, SD = 1.0).

#### Perceived discrimination

Experiences with everyday discrimination were assessed with a five-item measure on a four-point scale [47] where 0 = “Never,“ 1 = “Rarely,“ and 2 = “Sometimes”; responses of Often,“ “At least once a week,“ and “Almost every day” were categorized as 3. Participants were asked how often they are treated with less courtesy or respect than others, how often they receive poorer services at restaurants or stores, how often people act as if they are afraid of them, how often people act as if they are not smart, and how often they are threatened or harassed. Perceived discrimination was calculated as the mean score of these items (α = 0.91, M = 1.3, SD = 1.0).

#### Sociodemographic characteristics

Participant information about age, gender, race, education, marital status, living situation (live alone/live with someone), income, and overall health status was also obtained.

#### COVID-19 mitigation beliefs

On a 5-point scale where 1 = “Strongly disagree” and 5 = “Strongly agree,“ participants responded to two items asking how important they thought it was to wear a mask and maintain social distance when going out in public. These two items were combined for a mean score (α = 0.81, M = 4.5, SD = 1.0).

### Analysis plan

Multivariable logistic regression models were fitted for the first source of COVID-19 information (mass media vs. interpersonal) and the most trusted source (mass media vs. interpersonal), respectively. Specifically, an odds ratio (OR) larger than 1 indicated higher odds of choosing a mass media source, and an OR smaller than 1 showed higher odds of selecting an interpersonal information source. Univariable logistic regressions were fitted first with factors identified as potentially relevant to COVID-19 information-seeking based on previous research (e.g., [12, 29, 42, 48, 49]), and factors with *p*-values less than 0.15 were then considered for multivariable logistic regressions. Backward selection was used to build final multivariable models. Age, race, gender, education, marital status, and overall health status were kept in the multivariable model of the first source of COVID-19 information, while living situation, income, and marital status were kept in the multivariable model of most trusted source of COVID-19 information regardless of their *p*-values.

Multivariable multinomial logistic regression models were also fitted for the first sought and most trusted source of COVID-19 information to look at specific associations between source types, but in a non-aggregated fashion: comparing trusted individuals vs. Internet vs. printed materials vs. social media vs. Television vs. health care providers.

## Results

Participant characteristics are presented in Table 2. Average age was about 47 years (M = 46.6, SD = 17.5) with slightly more females (51.1%) than males (48.3%). Participants were primarily White (75.5%), followed by Black (14.8%) and Asian (5.8%). Most participants were college-educated (72.4%). In addition, a majority of participants were non-Hispanic (82.4%). Over half of the participants reported an income of $50,000 or greater (56%). Most participants were married (56.7%), living with someone else (77.6%), and did not live in a rural area (69.2%). Among the overall sample, 61.4% of participants preferred mass media as the first source of COVID-19 information, while the most trusted source was evenly split.

**Table 2:** Demographic characteristics of respondents (*n* = 1800)

Characteristics	*n* ^a^	%
Age	Mean 46.6	SD 17.5
Gender		
Male	865	48.3
Female	915	51.1
Other	12	0.7
Education		
High school or less	490	27.6
College or more	1288	72.4
Living Situation		
Live with someone	1376	77.6
Live alone	398	22.4
Income		
$0 to $19,999	301	17.6
$20,000 to $49,000	451	26.4
$50,000 to $99,999	500	29.3
$100,000 or more	455	26.7
Ethnicity		
Hispanic, Latino/a, or Spanish or Spanish Origin	313	17.6
NOT Hispanic, Latino/a, or Spanish Origin	1462	82.4
Race		
White	1293	75.5
Black/African American	254	14.8
Asian/Pacific Islander	100	5.8
American Indian	24	1.4
Other	42	2.5
Rural Residence		
Have lived in a rural or farming community or on a farm	518	30.7
NEVER lived in a rural or farming community or on a farm	1163	69.2
Marital Status		
Single, never been married	460	25.9
Married	1008	56.7
Divorced/Separated	208	11.7
Widowed	103	5.8
Self-Reported Overall Health Status		
Excellent	373	20.9
Very good	545	30.6
Good	555	31.1
Fair	261	14.6
Poor	49	2.7
Distribution of COVID-19 information-seeking		
First sought		
Interpersonal	635	38.6
Mass media	1010	61.4
Most trusted		
Interpersonal	803	49.6
Mass media	817	50.4

^a^Values do not always add up to *n* = 1800 due to missing values

### RQ1: How are sociodemographic characteristics associated with information seeking about COVID-19?

Table 3 presents univariable and multivariable logistic regression estimates for the association between individual characteristics and COVID-19 information-seeking behavior (See Additional file 2: Appendix for boxplots and bar graphs of significant predictors). Tables 4 and 5 present multivariable multinomial logistic regression estimates that provide a more granulated analysis of information-seeking across source category.

**Table 3:** Univariable and multivariable logistic regression models for first sought/most trusted sources for COVID-19 information

**Characteristics**	Univariable			Multivariable		
	OR^a^	95% CI	*P*	OR^a^	95% CI	*P*
**First sought**						
*** Age***	**1.02**	**1.01–1.02**	**< 0.01**	**1.02**	**1.01–1.03**	**< 0.01**
*** Covid Mitigation Strategy***	**1.27**	**1.14–1.41**	**< 0.01**	**1.21**	**1.07–1.36**	**< 0.01**
*** Race***			0.31			0.31
** African American v White (ref)**	**0.63**	**0.48–0.84**	**< 0.01**	0.76	0.55–1.06	0.11
** Asian v. White (ref)**	0.73	0.48–1.12	0.15	0.72	0.45–1.15	0.17
** Other v. White (ref)**	0.91	0.47–1.84	0.79	1.07	0.5202.27	0.85
*** Gender***			**< 0.01**			**0.03**
** Male v. Female (ref)**	**0.73**	**0.59**	**< 0.01**	**0.75**	**0.59–0.96**	**0.02**
** Other v. Female (ref)**	2.70	0.70-17.62	0.20	2.45	0.55–17.21	0.28
*** Health Status***			**0.05**			0.71
** Very good v. Excellent (ref)**	1.11	0.84–1.46	0.46	0.86	0.63–1.18	0.35
** Good v. Excellent (ref)**	**1.31**	**0.99–1.73**	**0.06**	0.86	0.61–1.2	0.37
** Fair v. Excellent (ref)**	**1.49**	**1.06–2.10**	**0.02**	1.00	0.67–1.51	0.99
** Poor v. Excellent (ref)**	**1.99**	**1.04–4.03**	**0.05**	1.16	0.56–2.54	0.69
*** Marital Status***			**0.04**			**0.01**
** Married v. Divorced (ref)**	**0.64**	**0.46–0.90**	**0.01**	0.91	0.61–1.33	0.62
** Single, never married v. Divorced (ref)**	**0.64**	**0.44–0.92**	**0.02**	1.04	0.67–1.62	0.85
** Widowed v. Divorced (ref)**	**0.52**	**0.31–0.86**	**0.01**	**0.43**	**0.25–0.75**	**0.003**
*** Education, high school or less***	**0.79**	**0.63–0.99**	**0.04**	**0.70**	**0.54–0.90**	**0.01**
*** Perceived Discrimination***	**0.73**	**0.66–0.81**	**< 0.01**	**0.83**	**0.73–0.94**	**< 0.01**
*** Self-efficacy***	**0.87**	**0.78–0.97**	**0.01**	**0.77**	**0.68–0.68**	**< 0.01**
**Most Trusted**						
*** Living With Someone Else***	**0.74**	**0.59–0.94**	**0.02**	**0.74**	**0.55-1.00**	**0.05**
*** Income***			**0.01**			0.22
** $20,000 to $49,999 v. <$20,000 (ref)**	0.78	0.57–1.07	0.13	0.85	0.61–1.18	0.34
** $50,000 to $99,999 v. <$20,000 (ref)**	**0.66**	**0.48–0.89**	**0.01**	0.73	0.52–1.01	0.06
** $100,000 or more v. <$20,000 (ref)**	**0.60**	**0.44–0.82**	**< 0.01**	0.72	0.5–1.03	0.07
*** Marital Status***			**0.03**			0.09
** Married v. Divorced (ref)**	**0.70**	**0.51–0.97**	**0.03**	0.92	0.63–1.34	0.67
** Single, never married v. Divorced (ref)**	0.97	0.68–1.38	0.87	0.95	0.65–1.38	0.79
** Widowed v. Divorced (ref)**	**0.51**	**0.31–0.84**	**0.01**	**0.54**	**0.32–0.9**	**0.02**
*** Perceived Discrimination***	1.03	0.93–1.13	0.60			
*** Self-efficacy***	**0.79**	**0.79–0.88**	**< 0.01**	**0.82**	**0.73–0.91**	**< 0.01**

^a^OR > 1 indicates higher odds of choosing Mass Media; OR < 1 indicates lower odds of choosing Mass Media (higher odds of choosing Interpersonal)

**Table 4:** Multivariable multinomial logistic regressions for first sought/most trusted sources for COVID-19 information

**Characteristics**	People You Trust vs. Health Care Providers		
	OR^a^	95% CI	***P***
**First sought**			
*** Age***	1.00	0.98–1.01	0.70
*** Covid-19 Mitigation Beliefs***	**0.66**	**0.55–0.8**	**< 0.01**
*** Race***			
African American v White (ref)	1.30	0.73–2.31	0.37
** Asian v. White (ref)**	**0.27**	**0.08–0.95**	**0.04**
Other v. White (ref)	1.81	0.52–6.32	0.35
*** Gender***			
Male v. Female (ref)	1.04	0.64–1.7	0.87
*** Health Status***			
Very good v. Excellent (ref)	1.12	0.6–2.08	0.72
Good v. Excellent (ref)	1.20	0.54–2.07	0.88
Fair v. Excellent (ref)	1.27	0.63–2.31	0.58
Poor v. Excellent (ref)	0.60	0.11–3.21	0.55
*** Marital Status***			
Married v. Divorced (ref)	1.21	0.48–3.05	0.68
Single, never married v Divorced (ref)	1.14	0.42–3.08	0.79
Widowed v. Divorced (ref)	2.49	0.8–7.77	0.12
* Education, high school or less*	1.05	0.64–1.74	0.82
*** Perceived Discrimination***	**1.30**	**1-1.68**	**0.05**
* Self-efficacy*	0.89	0.69–1.15	0.36
**Most Trusted**			
* Living With Someone Else*	1.25	0.67–2.3	0.48
*** Income***			
$20,000 to $49,999 v. <$20,000 (ref)	0.86	0.42–1.76	0.68
$50,000 to $99,999 v. <$20,000 (ref)	1.21	0.61–2.39	0.58
$100,000 or more v. <$20,000 (ref)	1.95	0.96–3.96	0.06
*** Marital Status***			
Married v. Divorced (ref)	0.96	0.43–2.17	0.93
Single, never married v Divorced (ref)	1.44	0.64–3.23	0.38
Widowed v. Divorced (ref)	1.34	0.48–3.75	0.58
*** Self-efficacy***	**0.76**	**0.62–0.94**	**0.01**

Findings presented in Tables 4 and 5 are from the same multinomial logistic regression model

^a^OR > 1 indicates higher odds of choosing People You Trust; OR < 1 indicates lower odds of choosing People You Trust (higher odds of choosing Health Care Provider)

**Table 5:** Multivariable multinomial logistic regressions for first sought/most trusted sources for COVID-19 information

**Characteristics**	Internet vs. Health Care Providers			Printed Material vs. Health Care Providers			Social Media vs. Health Care Providers			Television vs. Health Care Providers		
	OR^a^	95% CI	*P*	OR^a^	95% CI	***P***	OR^a^	95% CI	***P***	OR^a^	95% CI	***P***
**First sought**												
* Age*	1.01	1-1.02	0.14	**1.02**	**1-1.04**	**0.04**	0.99	0.98–1.01	0.57	**1.04**	**1.03–1.06**	**< 0.01**
*** Covid-19 Mitigation Beliefs***	1.03	0.88–1.2	0.72	1.04	0.82–1.31	0.76	1.20	0.93–1.55	0.17	1.22	0.98–1.53	0.07
*** Race***												
African American v White (ref)	0.69	0.44–1.07	0.10	0.63	0.31–1.29	0.21	1.59	0.9–2.83	0.11	1.00	0.58–1.72	0.99
Asian v. White (ref)	0.79	0.47–1.35	0.39	**0.22**	**0.05–0.95**	**0.04**	1.20	0.53–2.71	0.66	**0.11**	**0.03–0.49**	**< 0.01**
Other v. White (ref)	1.74	0.72–4.17	0.22	0.98	0.2–4.79	0.98	0.80	0.16–3.98	0.78	0.71	0.15–3.44	0.67
*** Gender***												
Male v. Female (ref)	**0.70**	**0.52–0.94**	**0.02**	1.23	0.76–2.02	0.40	0.84	0.51–1.37	0.48	**0.69**	**0.48–0.98**	**0.04**
*** Health Status***												
Very good v. Excellent (ref)	1.28	0.85–1.93	0.92	**0.41**	**0.24–0.69**	**< 0.01**	**0.49**	**0.28–0.88**	**0.02**	**2.12**	**1.21–3.73**	**0.01**
Good v. Excellent (ref)	1.30	0.84-2	0.24	**0.35**	**0.19–0.66**	**< 0.01**	**0.44**	**0.23–0.83**	**0.02**	**2.33**	**1.31–4.13**	**< 0.01**
Fair v. Excellent (ref)	**1.79**	**1.07–2.99**	**0.03**	0.46	0.21–1.02	0.06	0.56	0.25–1.25	0.16	**1.98**	**1.01–3.88**	**0.05**
Poor v. Excellent (ref)	1.62	0.64–4.11	0.31	0.53	0.13–2.13	0.37	0.80	0.19–3.31	0.75	2.42	0.8–7.31	0.12
*** Marital Status***												
Married v. Divorced (ref)	0.97	0.6–1.58	0.92	0.71	0.33–1.53	0.38	1.34	0.52–3.49	0.55	1.34	0.52–3.49	0.55
Single, never married v. Divorced (ref)	1.26	0.73–2.2	0.41	0.72	0.29–1.75	0.46	1.11	0.39–3.15	0.84	1.11	0.39–3.15	0.84
Widowed v. Divorced (ref)	0.62	0.31–1.26	0.19	0.36	0.09–1.44	0.15	0.24	0.03–2.17	0.20	0.24	0.03–2.17	0.20
*** Education, high school or less***	**0.50**	**0.36–0.71**	**< 0.01**	0.61	0.35–1.08	0.09	0.99	0.59–1.67	0.97	0.93	0.64–1.35	0.70
*** Perceived Discrimination***	**0.70**	**0.59–0.82**	**< 0.01**	**1.49**	**1.18–1.88**	**< 0.01**	1.26	0.98–1.62	0.07	**0.63**	**0.5–0.79**	**< 0.01**
*** Self-efficacy***	**0.71**	**0.6–0.84**	**< 0.01**	**0.88**	0.68–1.15	0.36	**0.66**	**0.52–0.86**	**0.02**	**0.73**	**0.6–0.89**	**< 0.01**
**Most Trusted**												
* Living With Someone Else*	0.78	0.53–1.14	0.19	**0.47**	**0.25–0.88**	**0.02**	0.98	0.51–1.89	0.96	0.85	0.55–1.33	0.48
*** Income***												
$20,000 to $49,999 v. <$20,000 (ref)	0.86	0.57–1.31	0.49	0.92	0.42–2.04	0.84	0.80	0.4–1.61	0.54	0.80	0.5–1.28	0.35
$50,000 to $99,999 v. <$20,000 (ref)	0.78	0.51–1.19	0.26	1.21	0.56–2.58	0.63	0.63	0.31–1.29	0.21	0.63	0.39–1.02	0.06
$100,000 or more v. <$20,000 (ref)	0.78	0.49–1.24	0.29	**2.18**	**1.02–4.69**	**0.05**	1.33	0.65–2.74	0.44	0.28	0.15–0.51	< 0.01
*** Marital Status***												
Married v. Divorced (ref)	0.88	0.54–1.43	0.60	**4.31**	**1.4–13.3**	**0.01**	4.13	0.93–18.25	0.06	0.54	0.32–0.9	0.02
Single, never married v. Divorced (ref)	1.15	0.72–1.84	0.57	**2.98**	**0.98–9.04**	**0.05**	**7.88**	**1.84–33.85**	**< 0.01**	0.41	0.24–0.68	< 0.01
Widowed v. Divorced (ref)	0.61	0.31–1.2	0.15	1.47	0.56–2.58	0.63	1.46	0.2-10.78	0.71	0.40	0.2–0.82	0.01
*** Self-efficacy***	**0.72**	**0.63–0.83**	**< 0.01**	0.94	0.75–1.19	0.62	**0.67**	**0.53–0.83**	**< 0.01**	0.85	0.72–1.01	0.07

Findings presented in Tables 4 and 5 are from the same multinomial logistic regression model

^a^OR > 1 indicates higher odds of choosing Mass Media category; OR < 1 indicates lower odds of choosing Mass Media category (higher odds of choosing health care provider)

Tables 3, 4 and 5 also present univariable and multivariable logistic regression estimates along with findings from the multinomial analysis to evaluate the association between individual characteristics and COVID-19 information-seeking behavior.

#### Univariable/multivariable logistic model

On univariable analysis, characteristics associated with a preference for mass media as the first source of information rather than interpersonal connections were older age (OR: 1.02, *p* < .01), poor health status (OR: 1.99, *p* = .05), and stronger beliefs in the importance of masking and social distancing (OR: 1.27, *p* < .01). Conversely, factors related to a preference for interpersonal communication as an initial source were self-identifying as Black or African American (OR: 0.63, *p* = < 0.01), self-identifying as male (OR: 0.73, *p* = < 0.01), and high school education or less (OR: 0.79, *p* = .04). Further, related to trustworthiness, living with someone else (OR: 0.74, *p* = .02). Having a higher income level (see Table 3) was associated with greater trust in interpersonal sources of COVID-19 information in the univariable model.

In the multivariable model, older age and stronger beliefs in the importance of masking and social distancing were independently associated with a preference for mass media as the first source of COVID-19 information. Self-identifying as male and less educational attainment were independently related to increased odds of seeking COVID-19 information first from interpersonal sources. Living with someone else was independently associated with trust in interpersonal rather than mass media sources.

#### Multivariable multinomial logistic model

Findings from the multivariable multinomial analysis suggest the preference of older adults for mass media as a first source of information was only significant for printed materials (e.g., newspapers, magazines) (OR: 1.02, *p* = .04) and television (OR: 1.04, *p* < .01) when compared to health care providers. There was no specific preference for mass media type based on mitigation beliefs. Also, while there was not a reported preference for interpersonal source based on educational attainment, the Internet (e.g., Google, WebMD) was less preferred as an initial source of COVID-19 information by participants with less formal educational attainment when compared to health care providers (OR: 0.50, *p* < .01). Similarly, while males were inclined towards interpersonal sources first, there was not a meaningful difference in the preferred interpersonal source type based on gender. However, male participants did report less preference for Internet (OR: 0.70, *p* = .02) and television (OR: 0.69, *p* = .04) sources when compared to their health care providers.

Regarding the sources most trusted for COVID-19 information, living with someone else was not found to have a significant relationship with a preferred interpersonal source, but printed materials were considered a less trustworthy source of information compared to health care providers for individuals living with another person (OR: 0.47, *p* = .02).

### RQ2: How are discrimination and self-efficacy associated with information-seeking about COVID-19?

Tables 3, 4 and 5 also present univariable and multivariable logistic regression estimates for the relationship between self-efficacy, perceived discrimination, and COVID-19 information-seeking behavior.

#### Univariable/multivariable logistic model

On univariable analysis, experiences with discrimination (OR: 0.73, *p* < .01) were related to a preference for interpersonal sources of COVID-19 information. Further, greater confidence in personal health information-seeking ability (self-efficacy) was associated with seeking out interpersonal sources first (OR: 0.87, *p* = .01) and regarding these sources as more trustworthy (OR: 0.79, *p* < .001) compared to mass media sources.

In the multivariable model, having more experiences with discrimination was independently related to an increased odds of seeking COVID-19 information first from interpersonal sources. Increased self-efficacy was also an independent correlate of both increased preference and trust in interpersonal sources for COVID-19 information compared to mass media.

#### Multivariable multinomial logistic model

Results of the multivariable multinomial analysis suggest that individuals with stronger experiences with discrimination preferred to seek out COVID-19 information first from trusted family, relatives, or coworkers (OR: 1.30, *p* = .05) and printed materials (OR: 1.5, *p* < .01), but were less likely to seek information first from the Internet (OR: 0.70, *p* < .01) and television (OR: 0.63, *p* < .01) compared to their health care provider. There was not a meaningful difference in which interpersonal source participants preferred based on self-efficacy; however, greater efficacy was associated with less preference for the Internet (OR: 0.71, *p* < .01), social media (OR: 0.66, *p* = .02), and television (OR: 0.73, *p* < .01) compared to health care providers.

Regarding the most trusted source of COVID-19 information, individuals with greater efficacy had smaller odds of viewing their family, relatives, or coworkers (OR: 0.76, *p* = .01), Internet (OR: 0.72, *p* < .01), and social media (OR:0.67, *p* < .01) as a trustworthy source of information compared to health care providers. Table 6 provides a summary of the study findings.

**Table 6:** Summary of main findings

Variable	Univariable	Multivariable	Multinomial
**FIRST SOUGHT**			
Age	Older (+) mass media	Older (+) mass media	Older (+) print & TV
COVID-19 mitigation beliefs	Stronger beliefs (+) mass media	Stronger beliefs (+) mass media	Not significant
Race	Black adults (+) interpersonal	Not significant	
Gender	Male (+) interpersonal	Male (+) interpersonal	Male (-) Internet & TV
Education	Less educational attainment (+) interpersonal	Less educational attainment (+) interpersonal	Less educational attainment (-) Internet
Discrimination	Stronger discrimination (+) interpersonal	Stronger discrimination (+) interpersonal	Stronger discrimination (+) friends/ relatives/co-workers & print; (-) Internet & TV
Self-efficacy	Greater efficacy (+) interpersonal	Greater efficacy (+) interpersonal	Greater efficacy (-) Internet, social media, & TV
**MOST TRUSTED**			
Living with someone	Living with someone (+) interpersonal	Living with someone (+) interpersonal	Living with someone (-) print
Self-efficacy	Greater efficacy (+) interpersonal	Greater efficacy (+) interpersonal	Greater efficacy (-) friends/relatives/coworkers, Internet, and social media

## Discussion

The purpose of this study was to explore factors associated with audience preferences (first sought, most trusted) for COVID-19 information to inform the development of tailored health communication strategies. The current work adds to literature on HISB during the COVID-19 pandemic by providing evidence for the relationship between sociodemographics and source trust first proposed by Ali et al. [41], and extends by demonstrating how information sources, notably those first sought, are related to discrimination and information efficacy.

### Sociodemographics driving COVID-19 information-seeking

#### Age

One key finding is that mass media outlets, specifically print materials (e.g., newspapers, magazines) and TV, were preferred as initial sources for COVID-19 information for older participants. The elderly are particularly vulnerable to becoming severely ill from COVID-19, increasing the urgency for tailored communication strategies [50]. This preference for mass media as initial sources of information conflicts with previous findings suggesting that older adults rely on interpersonal sources such as health care providers and family members, not only for information but also to satisfy emotional needs stemming from social isolation during the pandemic [51, 52].

One rationale for this inconsistency might be that the COVID-19 pandemic morphed into a political wedge issue in which risk perceptions, conspiracy beliefs, and responses to government recommendations were demarcated along partisan lines [6, 53, 54]. As a result, older adults might have sought information from their political echo chambers (e.g., cable news networks) rather than other sources such as government websites or health care providers [41, 55, 56].

Another explanation is that the novelty of the SARS-CoV-2 virus and the associated uncertainty, fear, and confusion limited the value of interpersonal discussions, prompting information to be sought elsewhere. It is worth noting that a large portion of this sample was college-educated, and other factors including health status may have contributed to this finding; individuals with chronic conditions may access COVID-19 information more often through the mass media but have less trust in these sources [57]. The interaction of age and health status on COVID-19 information-seeking is an area of future study.

#### Education

Another key finding was that communication with interpersonal sources was preferred as a primary resource for information by those with lower levels of educational attainment. Further analysis revealed that there was not a significant difference in preference of first information source for participants with less formal education between preferring friends/relatives/co-workers or health care providers. However, the Internet was a less preferred source compared to health care providers for these participants, suggesting that providers can be targeted for campaigns aimed at this group. Studies of education level and COVID-19 misinformation have reported relationships with a multitude of factors, including lower confidence in government and scientific institutions as well as lower perceived infection risk [58, 59]. However, previous research suggests that those with less formal education may perceive a greater risk of dying from COVID-19 and experience greater economic consequences because of the pandemic [58]; it is possible that this increased risk prompts information-seeking from professional sources. Further, individuals with lower levels of educational attainment are more likely to have reduced health literacy, and these individuals may instead turn to their doctors for information [60]. Educational attainment has been found to positively correlate with a diversity of sources [43], furthering the argument that education level is a barrier to information-seeking through mass media.

#### Mitigation beliefs

Participants with weaker beliefs in the importance of masking and social distancing when in public were more likely to seek out COVID-19 information through their interpersonal contacts first, regardless of the source. Individuals with strong doubts about the effectiveness of masking and social distancing are less prone to seek knowledge through external mass media channels, particularly when there is evolving information [61]. This finding offers confirming evidence for previous research demonstrating a significant relationship between COVID-19 information-seeking and adherence to mitigation strategies [62].

Given the politicization and polarization of the pandemic, those more skeptical of mitigation strategies would be more likely to look for information within their interpersonal networks rather than a media system that is viewed as biased [26, 63, 64]. These individuals may be challenging to target with health communication campaigns. However, given the demonstrated direct relationship between COVID-19 information seeking and preventive behavior [65, 66], there is a pressing need for evidence-based efforts.

### Discrimination and self-efficacy driving COVID-19 information-seeking

#### Discrimination

Individuals reporting more common experiences with discrimination also described a greater preference for interpersonal contacts as an initial source for COVID-19 information, specifically friends, relatives, and co-workers. Discrimination can cause a delay in seeking medical care, including cancer screenings [67], and significantly increases stress response [68]. One speculation for this finding is that while mass media may be used as a means of coping with the stress that comes along with mistreatment, information exposure during a health crisis such as COVID-19 can intensify feelings of stress, leading to avoidance [69, 70]. Given the high levels of discrimination reported during the pandemic [71, 72], these groups may find it less distressing to receive information from trusted interpersonal sources, particularly those that share similar demographic backgrounds [69]. Additional research is needed to disentangle the effect of different sources of discrimination (e.g., gender, race, ethnicity) on information-seeking about COVID-19 [73].

#### Self-efficacy

Finally, this work also found that individuals with greater confidence in their ability to obtain health information preferred to seek out interpersonal sources first, with a particularly lower preference for the Internet, social media, and TV compared to their health care provider. Participants with greater efficacy also found interpersonal sources to be more trustworthy, yet maintained a lower perception of trustworthiness for friends, relatives, and family compared to health care providers. Individuals tend to make determinations on whether to engage in information-seeking by evaluating three types of efficacies: communication efficacy (whether the individual has the skill to seek information), target efficacy (whether their interpersonal source has the knowledge and is willing to share it), and coping efficacy (whether the individual can emotionally deal with the information) [21]. Thus, individuals with greater efficacy may feel more confidence in their ability to seek information from interpersonal sources based on their communication skills, beliefs that their interpersonal sources have reliable information, and beliefs that they can cope with the information potentially shared.

Interpersonal sources may also help calm the often overwhelming “noise” of competing and emerging information shared by media channels. Individuals who are confident in obtaining health information are also more likely to experience feelings such as fatalism when they experience challenges and frustrations in seeking this information [74]. Therefore, individuals with increased self-efficacy in their HISB may prefer to engage with interpersonal sources rather than mass media to attenuate the uncertainty associated with this massive influx of information.

## Practical implications

Audience segmentation refers to the process of dividing an audience into definable, measurable groups to create messaging that is responsive to specific population needs [75]. This approach to message design can significantly impact engagement, as well as attitude and behavior change [76] and is thus considered an essential piece of tailored communication strategies already applied to COVID-19 messaging [69].

Findings from this study have meaningful implications for future practice through the identified audience variations regarding information-seeking preferences. These results can be leveraged to enhance the capability of specific target audiences to engage with evidence-based COVID-19 information. The politicization of COVID-19 and its influence on health inequalities, along with the rapid and uneven pace of information dissemination on COVID-19 guidelines, has been a challenge for effective health communication [77]. Thus, health communication campaigns that can efficiently identify strategies to reach various audiences in a targeted manner will have increased effectiveness. The following guidelines should be priority considerations when developing audience-focused COVID-19 information campaigns:

1. Understand the unique contexts of the intended audience, including the influence of societal inequalities on information-seeking behavior. Taking a user-centered approach to campaign design that actively seeks out and incorporates feedback will ensure that the preferences, needs, and values of the target audience are fully understood. This approach will also enhance campaign acceptability while reducing the effort required to engage with its components, all of which will increase efficacy. We offer the following specific recommendations for campaign tailoring based on the findings of this study:
  Campaigns targeting older adults should develop materials for dissemination through television and print.
  When developing campaigns targeting individuals with less formal educational attainment, include medical professionals.
  Incorporating close social ties (i.e., friends, relatives, and co-workers) may increase the effectiveness of campaigns targeting groups experiencing discrimination.
  Audiences with greater efficacy can be effectively targeted through their health care provider, whereas those with weaker beliefs in their ability to obtain health information can be better reached through the Internet (e.g., WebMD) and social media.

2. In addition to examining the “what” and “how” of message dissemination, the “where” and “who” should also be carefully considered. Theoretical frameworks such as diffusion of innovations [78] and social influence [79] can serve as starting points to further understand the influence of social networks and source credibility in information-seeking. Building capacity to bring these campaigns to scale will also be required and can be facilitated through the development of diverse collaborations that include community members and other stakeholders.
3. Lastly, consider the context of the topic and understand that source preferences for information may vary when the topics change, particularly given the political climate (e.g., COVID-19 information seeking may be very different than cancer screening). Campaign development should be iterative and agile in order to adapt to the fluidity inherent to these politically charged health topics, with systems in place for ongoing evaluation.

### Strengths and limitations

This study adds to the literature on information-seeking about COVID-19 through the examination of sources of COVID-19 information most likely to be sought first and the exploration of the role of discrimination and self-efficacy on source preference (i.e., first sought and most trusted). The findings also offer support for previous research on the influence of sociodemographic factors in HISB.

This study is not without limitations. The measures of information sources may contain within-group differences (e.g., different social media platforms such as Facebook and Twitter are often used in different ways). Yet, this study provides compelling evidence for HISB during the pandemic and how individuals can be targeted with persuasive messaging. Also, while this online survey asked only for the FIRST preferred source or the MOST trusted, communication does not occur in a vacuum. Mass and interpersonal methods of communication are becoming increasingly intermingled [77], and factors such as authority (e.g., government websites and health care providers) might play a role [41]. Additional research is needed to build information-seeking models of increasing complexity surrounding the interplay of these factors.

## Conclusion

The COVID-19 pandemic has weakened the US economy and led to tremendous life loss, and the uptake of protective measures is lacking due in part to false information being circulated within the media and personal networks. The findings of this study contribute to our understanding of how people are seeking out information about COVID-19 during the pandemic, which will allow for the development of evidence-based dissemination strategies. As information-seeking increases during the pandemic, exposure to risk information can have a direct tie to behavior, and the results of this study suggest that even with such a wide diversity of digital information sources and the capacity for scalable health communication campaigns that maximizing efforts to involve interpersonal connections may be preferable for some individuals. This idea is even more relevant during the current infodemic, where mass media channels have, in many ways, been corrupted by misinformation. By considering the audience factors illuminated in this study, researchers and practitioners become better equipped to deliver messaging through the sources and channels that are highly sought and trusted.

## Supplementary Information

**Additional file 1.** Study questionnaire. 

**Additional file 2: Appendix 1.** Boxplots and bar graphs for predictors of COVID-19 information-seeking. 

## Data Availability

Data are available upon request from the corresponding author.
