# Supplementary material for: What did the pandemic teach us about effective health communication? Unpacking the COVID-19 infodemic
Source: BMC Public Health. 2022 Dec 13;22:2339. doi: 10.1186/s12889-022-14707-3 (PMC9747260; doi:10.1186/s12889-022-14707-3)
Supplement: Supplementary file 1 — Additional file 1. Study questionnaire. [file 12889_2022_14707_MOESM1_ESM.docx]

**Additional_file_1**

**Study questionnaire**

**COVID-19 Information-Seeking**

When you had a strong need to get information about COVID-19, where did you FIRST go to get information?

- Printed materials (for example, newspapers, magazines)
- Health care provider (doctor, nurse, social worker)
- Conversations with people you trust (friends, relatives, or co-workers)
- Internet (google or another search engine, WebMD or another medical website)
- Social media (Facebook, Instagram, twitter)
- Television
- Other _____________________
- Prefer not to answer

When you had a strong need to get information about COVID-19, which of following did you find to be the MOST trusted as a source of information about coronavirus or COVID-19?

- Printed materials (for example, newspapers, magazines)
- Health care provider (doctor, nurse, social worker)
- Conversations with people you trust (friends, relatives, or co-workers)
- Internet (google or another search engine, WebMD or another medical website)
- Social media (Facebook, Instagram, twitter)
- Television
- Other _________________________
- Prefer not to answer

**Perceived Discrimination**

In your day-to-day life, how often have any of the following things happened to you:

You are treated with less courtesy or respect than other people.

- - Never
  - Rarely
  - Sometimes
  - Often
  - At least once a week
  - Almost every day
  - Don’t know
  - Prefer not to answer

You receive poorer services than other people at restaurants and stores.

- - Never
  - Rarely
  - Sometimes
  - Often
  - At least once a week
  - Almost every day
  - Don’t know
  - Prefer not to answer

People act as if they are afraid of you.

- - Never
  - Rarely
  - Sometimes
  - Often
  - At least once a week
  - Almost every day
  - Don’t know
  - Prefer not to answer

People act as if they think you are not smart.

- - Never
  - Rarely
  - Sometimes
  - Often
  - At least once a week
  - Almost every day
  - Don’t know
  - Prefer not to answer

You are threatened or harassed.

- - Never
  - Rarely
  - Sometimes
  - Often
  - At least once a week
  - Almost every day
  - Don’t know
  - Prefer not to answer

**Self-efficacy**

Overall, how confident are you that you could get advice or information about health or medical topics if you needed it?

- - Completely confident
  - Very confident
  - Somewhat confident
  - A little confident
  - Not confident at all
  - Don't know
  - Prefer not to answer

**COVID-19 Mitigation Strategy**

COVID-19 can be spread to others even when you do not feel sick. Do you think that it is important to wear a face mask when going out in public?

- Strongly agree
- Somewhat agree
- Somewhat disagree
- Strongly disagree
- Neither
- Prefer not to answer​

COVID-19 can be spread to others even when you do not feel sick. Do you think that it is important to maintain social distance (about 6 feet between yourself and others) when going out in public?

- Strongly agree
- Somewhat agree
- Somewhat disagree
- Strongly disagree
- Neither
- Prefer not to answer​

**Concern about COVID-19**

Are you concerned about coronavirus or COVID-19 in your community? Please answer with a number between 0 and 10, 0 means NO concern about COVID-19 and 10 means VERY concerned. You can use ANY number, including fractions, between 0 and 10 to let us know how much concern you have about COVID-19 right now. __________

- Prefer not to answer

**Demographics**

What is your age? ____________

How do you describe your gender?

- Male
- Female
- Transgender
- Do not identify as female, male, or transgender
- Prefer not to answer

What is your race? *One or more categories may be selected.*

- American Indian
- Alaska Native
- Asian
- Black/African American
- Native Hawaiian/ Pacific Islander
- White
- Don't know
- Prefer not to answer

Are you Hispanic, Latino/a, or Spanish origin?

- Yes
- No
- Prefer not to answer

What is your current marital status?

- Married/Domestic Partner
- Living as married
- Divorced
- Widowed
- Separated
- Single, never been married
- Don't know
- Prefer not to answer

Have you ever lived in a rural or farming community, or on a farm?

- Yes, for how many years? ____
- No
- Prefer not to answer

What is your living situation?

- Live alone
- With spouse/partner
- With family members
- Assisted living facility
- Other:________
- Prefer not to answer

What is the highest grade or level of schooling you have completed?

- Less than 8 years
- 8 through 11 years
- 12 years or completed high school/GED
- Post high school training other than college (vocational or technical)
- Some college
- College graduate
- Postgraduate
- Don't know
- Prefer not to answer

For all members living in your household, what is the combined annual income (the total pre-tax income from all sources earned in the past year)?

- $0 to $9,999
- $10,000 to $19,999
- $20,000 to $34,999
- $35,000 to $49,999
- $50,000 to $74,999
- $75,000 to $99,999
- $100,000 or more
- Don't know
- Prefer not to answer

Would you say that in general your health is—?

- - Excellent
  - Very good
  - Good
  - Fair
  - Poor
  - Prefer not to answer
