# Supplementary figures and images for: What did the pandemic teach us about effective health communication? Unpacking the COVID-19 infodemic
Source: BMC Public Health. 2022 Dec 13;22:2339. doi: 10.1186/s12889-022-14707-3 (PMC9747260; doi:10.1186/s12889-022-14707-3)

**Appendix 1. Boxplots and bar graphs for predictors of COVID-19 information-seeking**


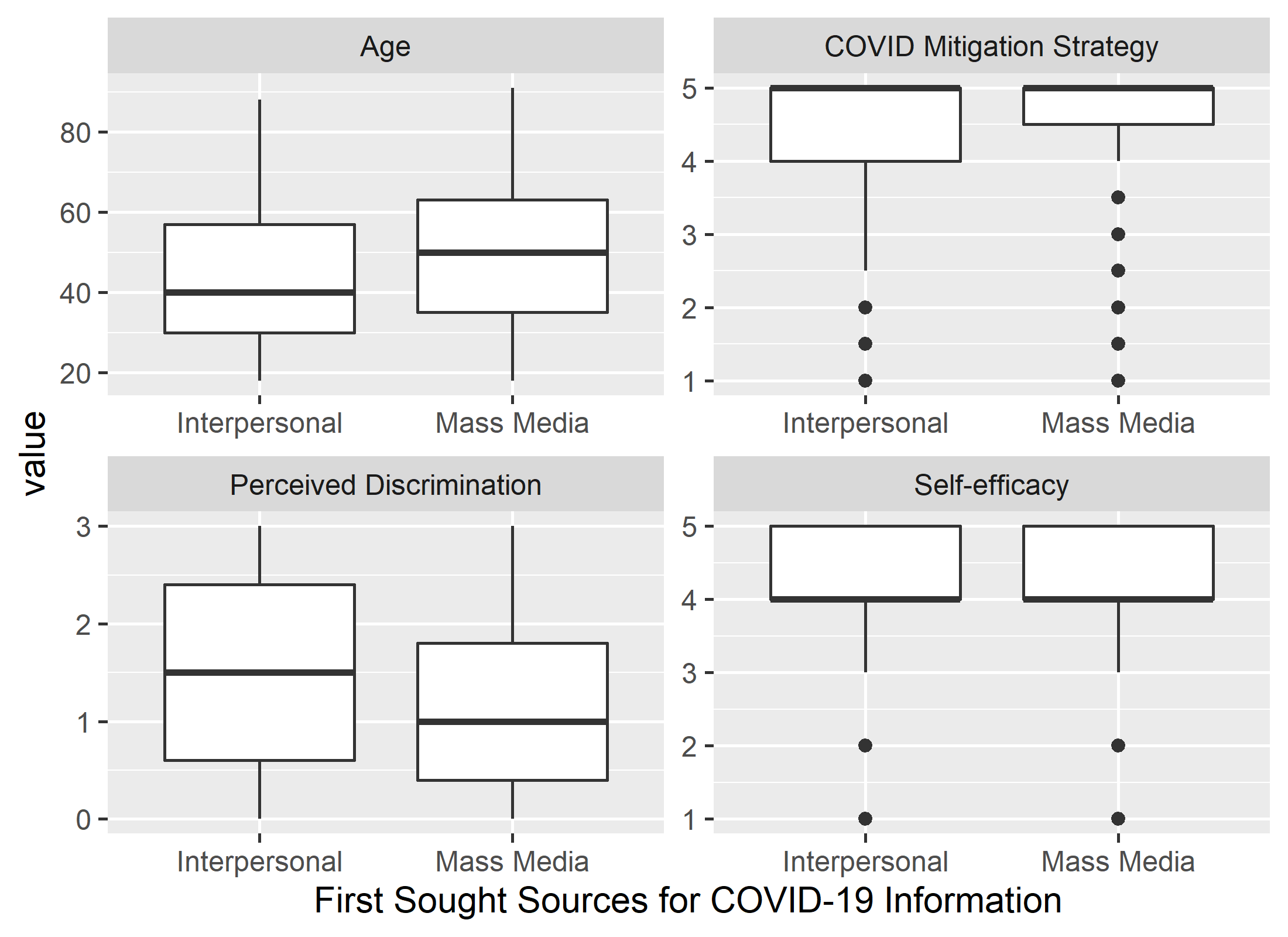


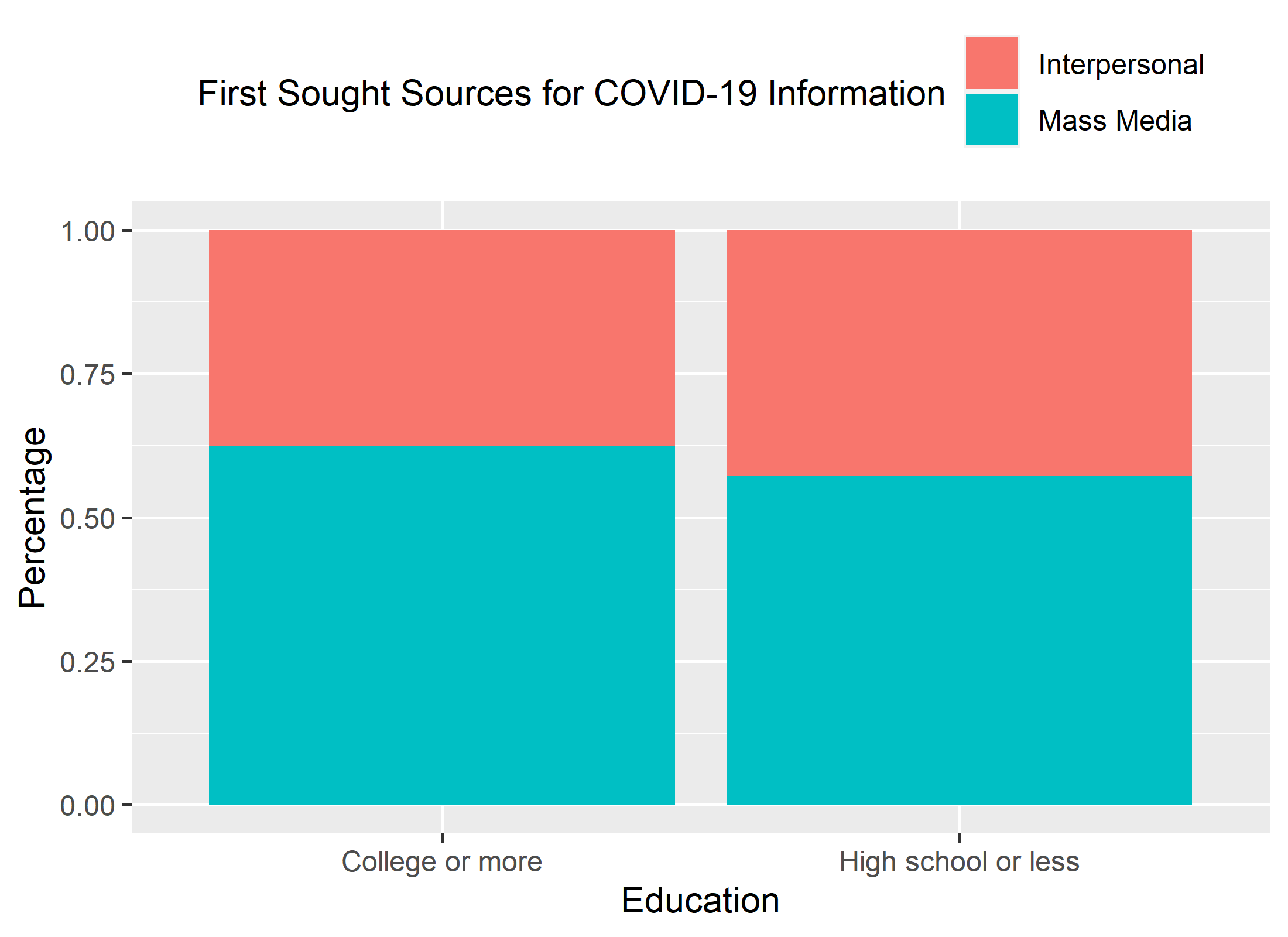


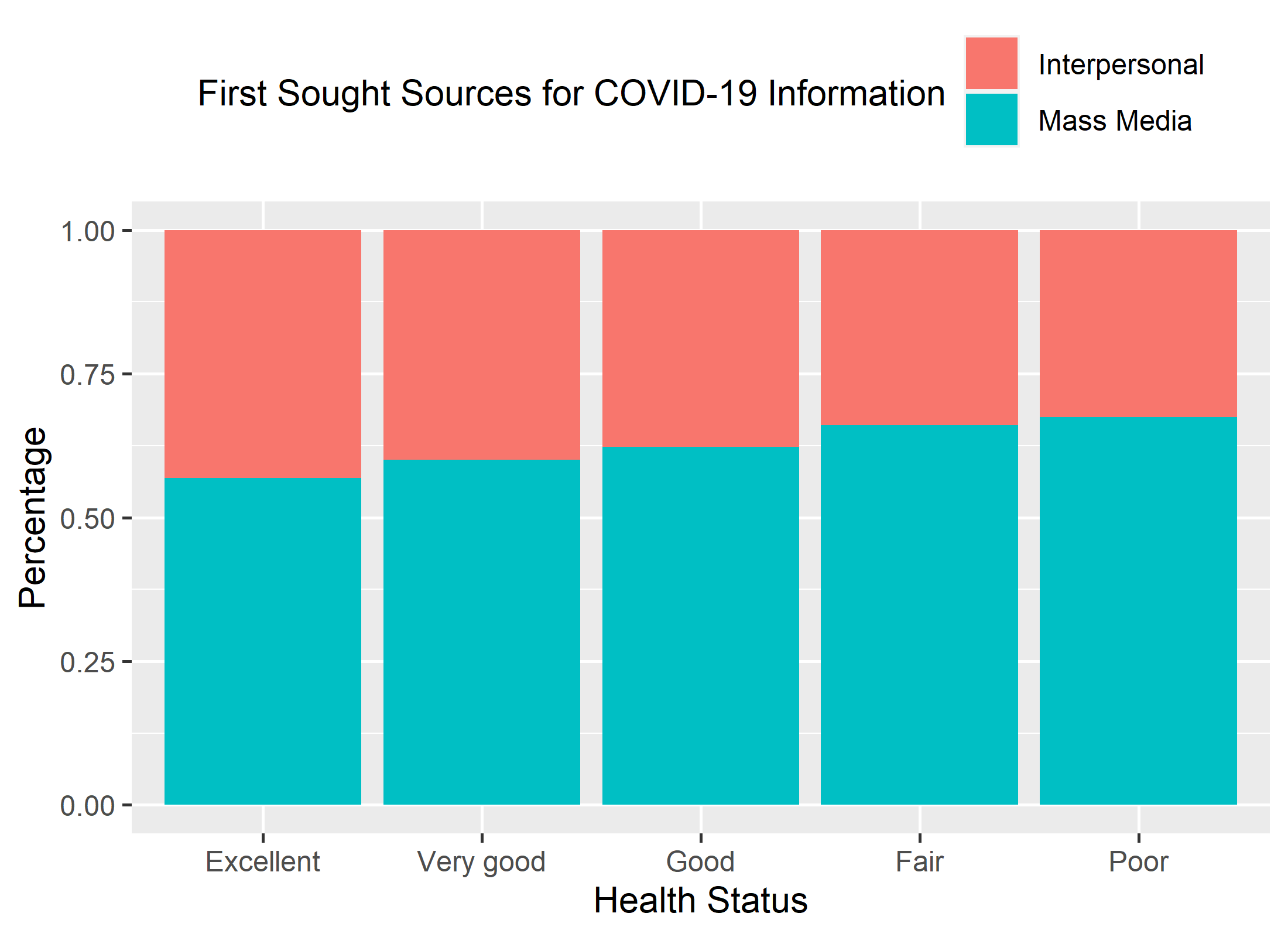


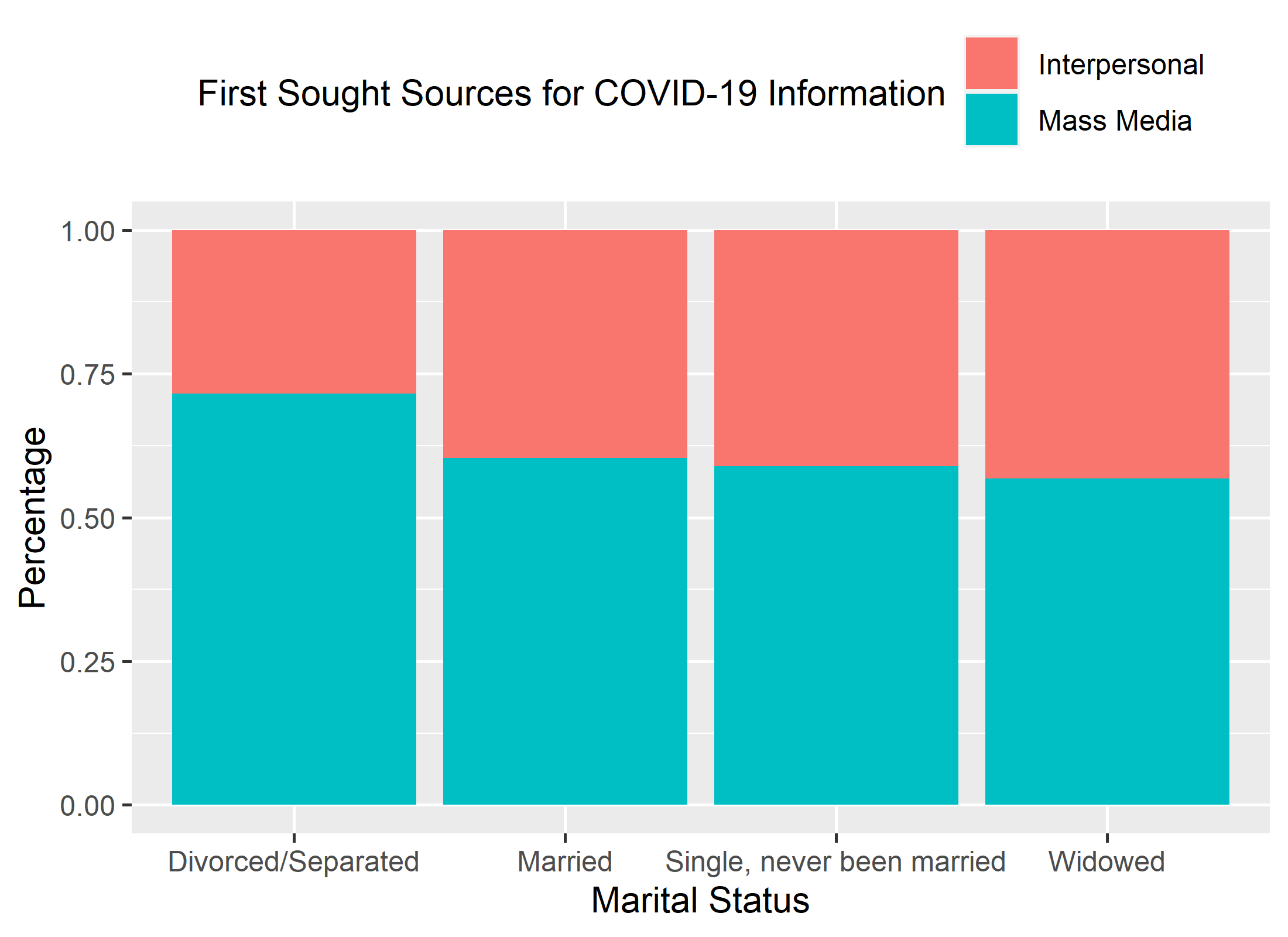


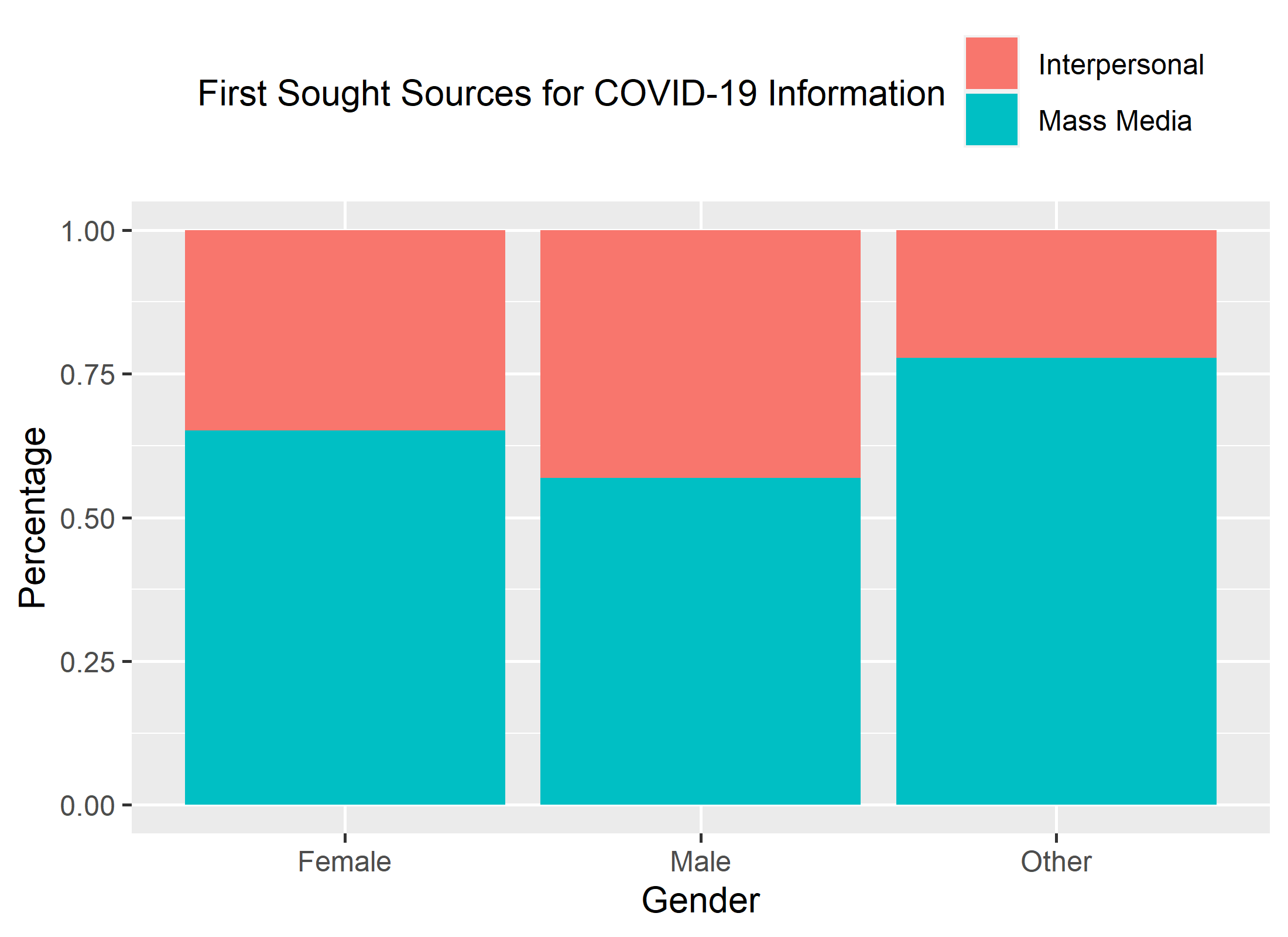


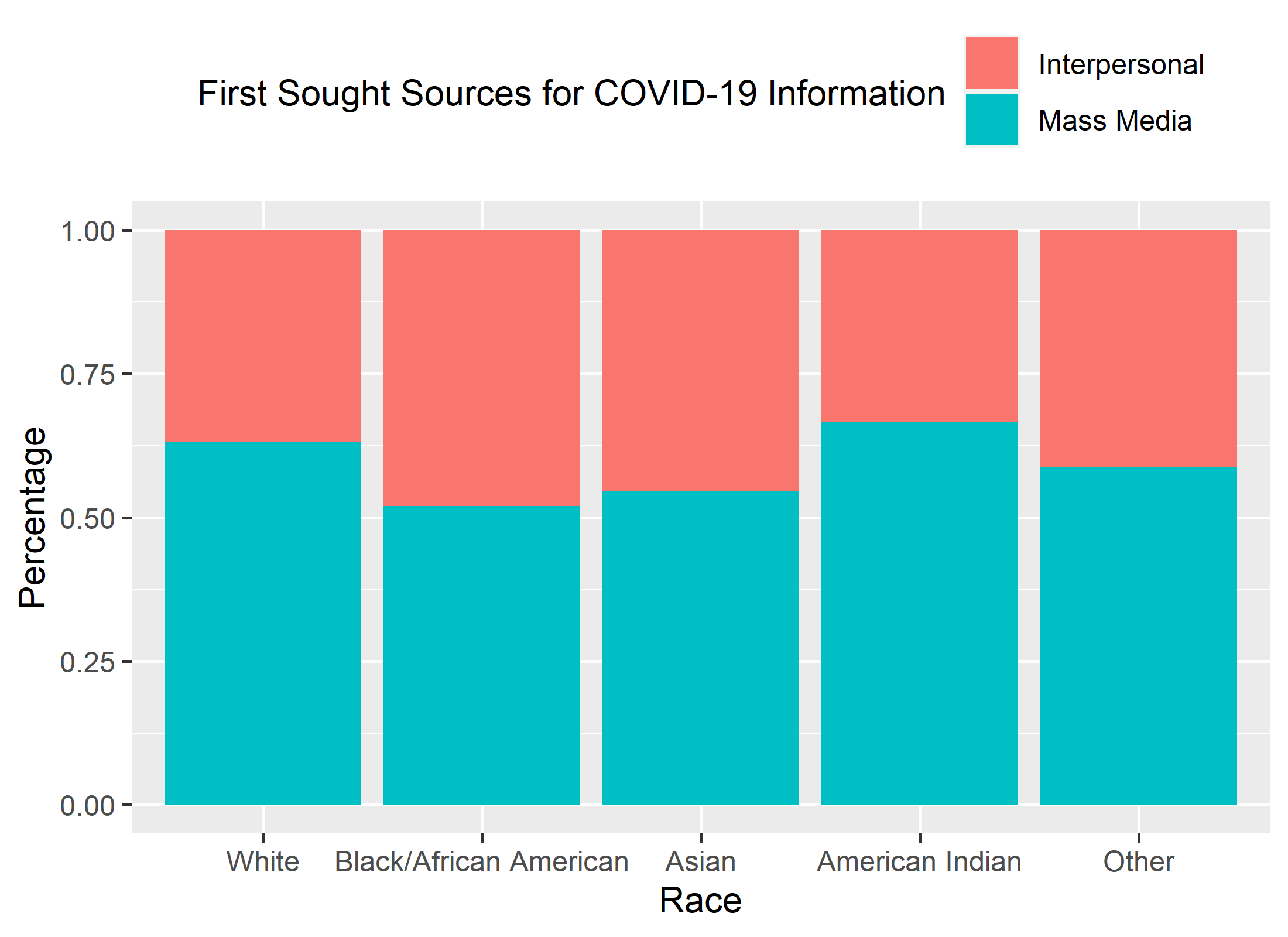


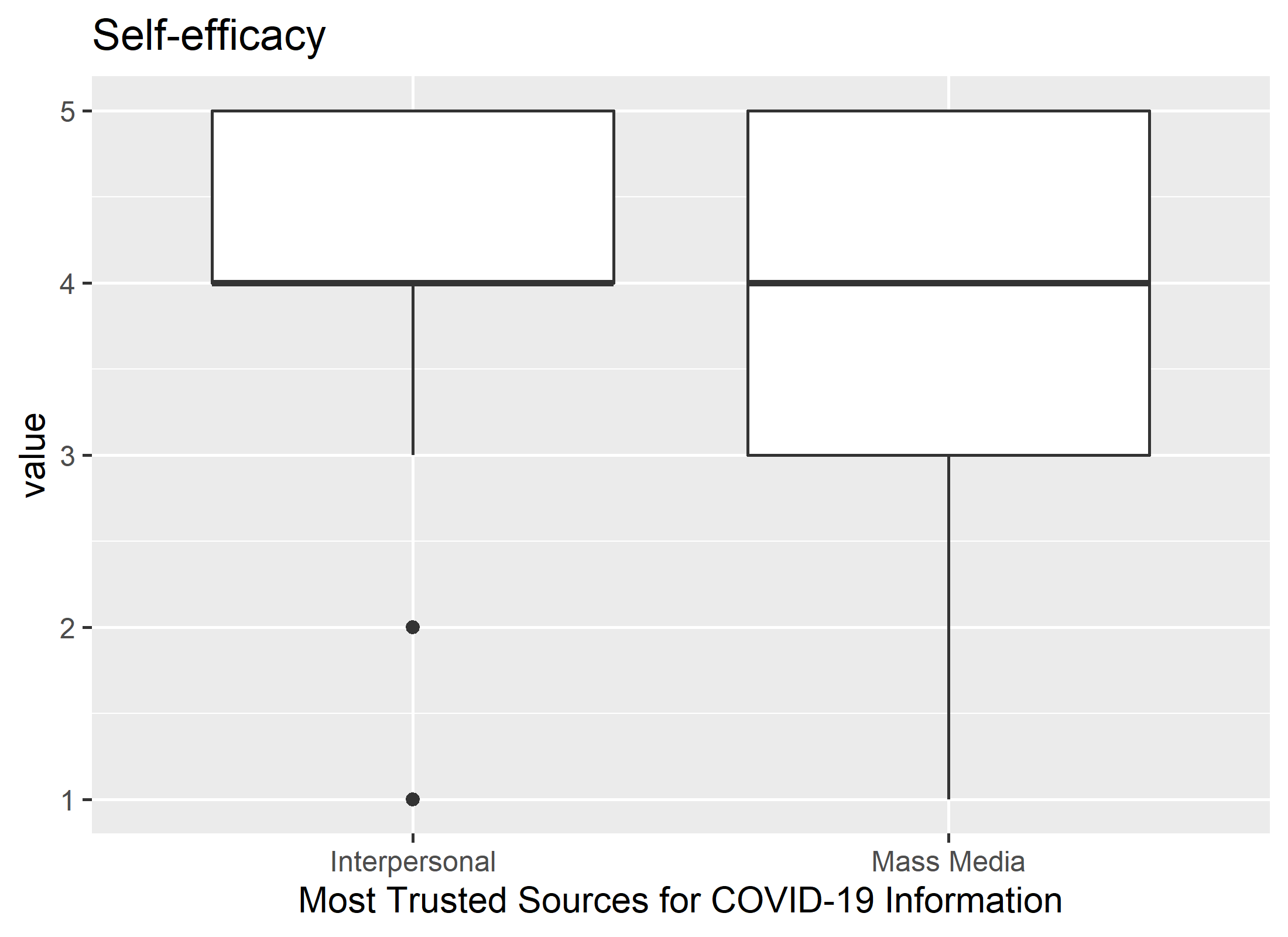


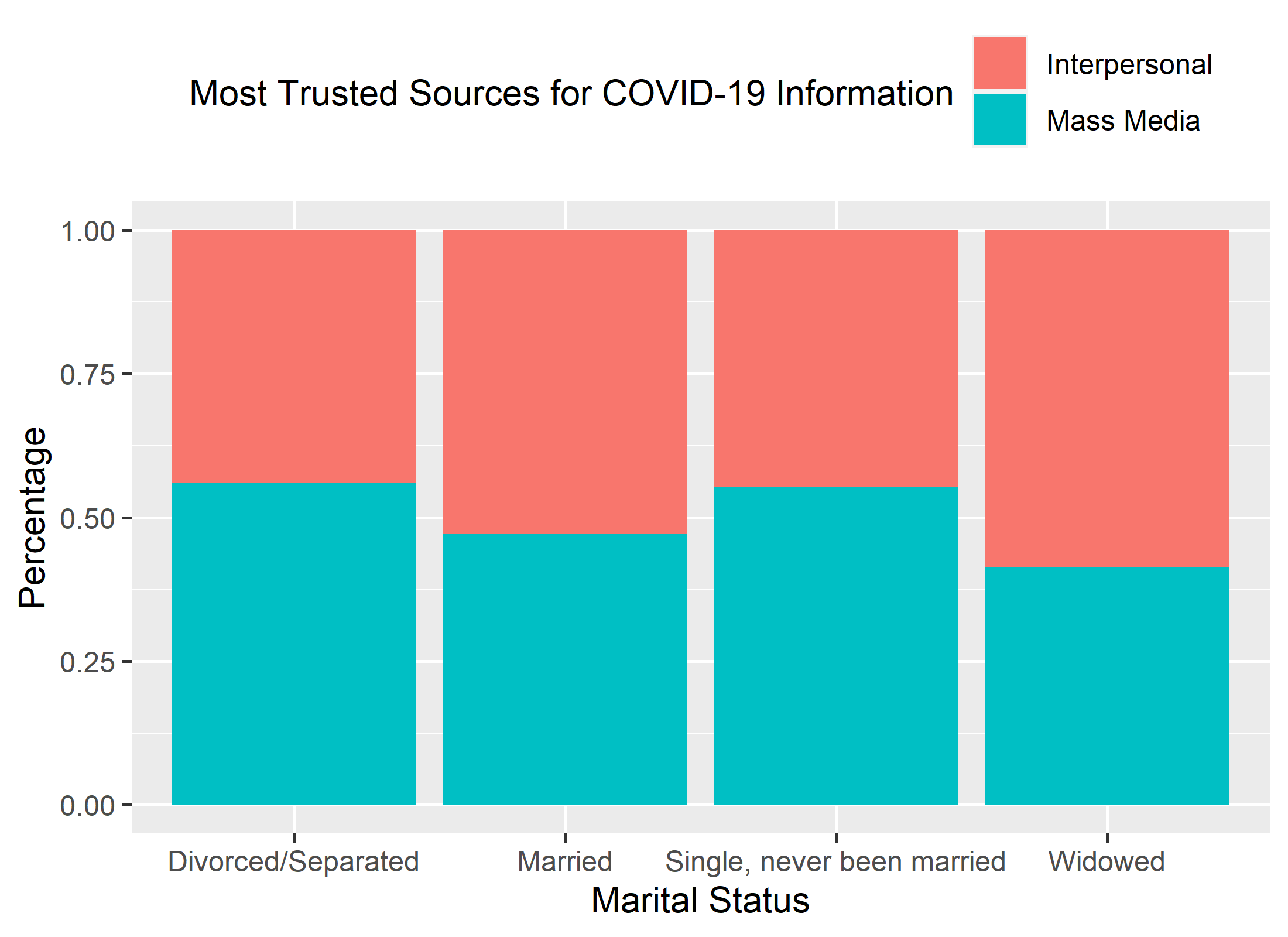


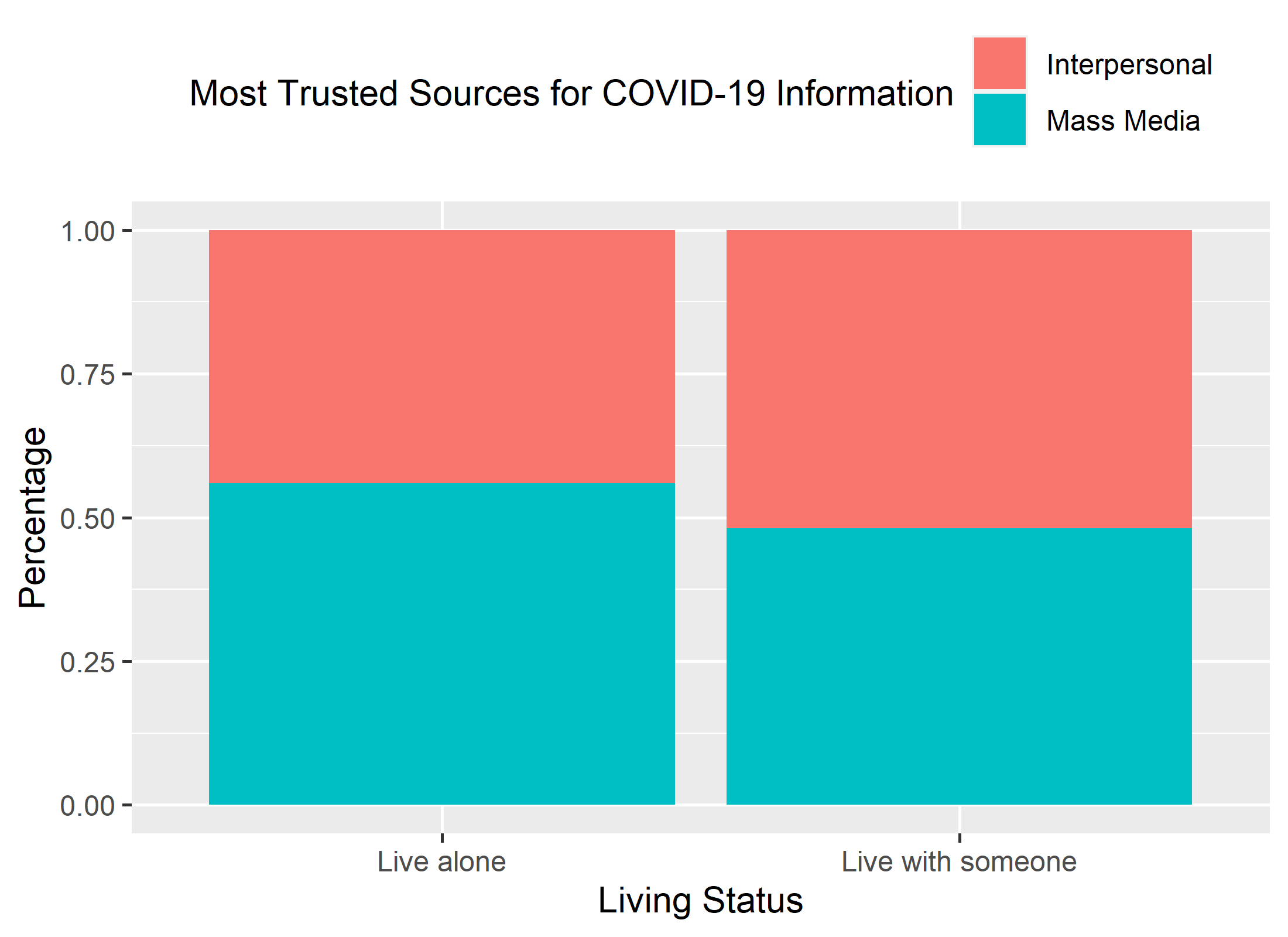


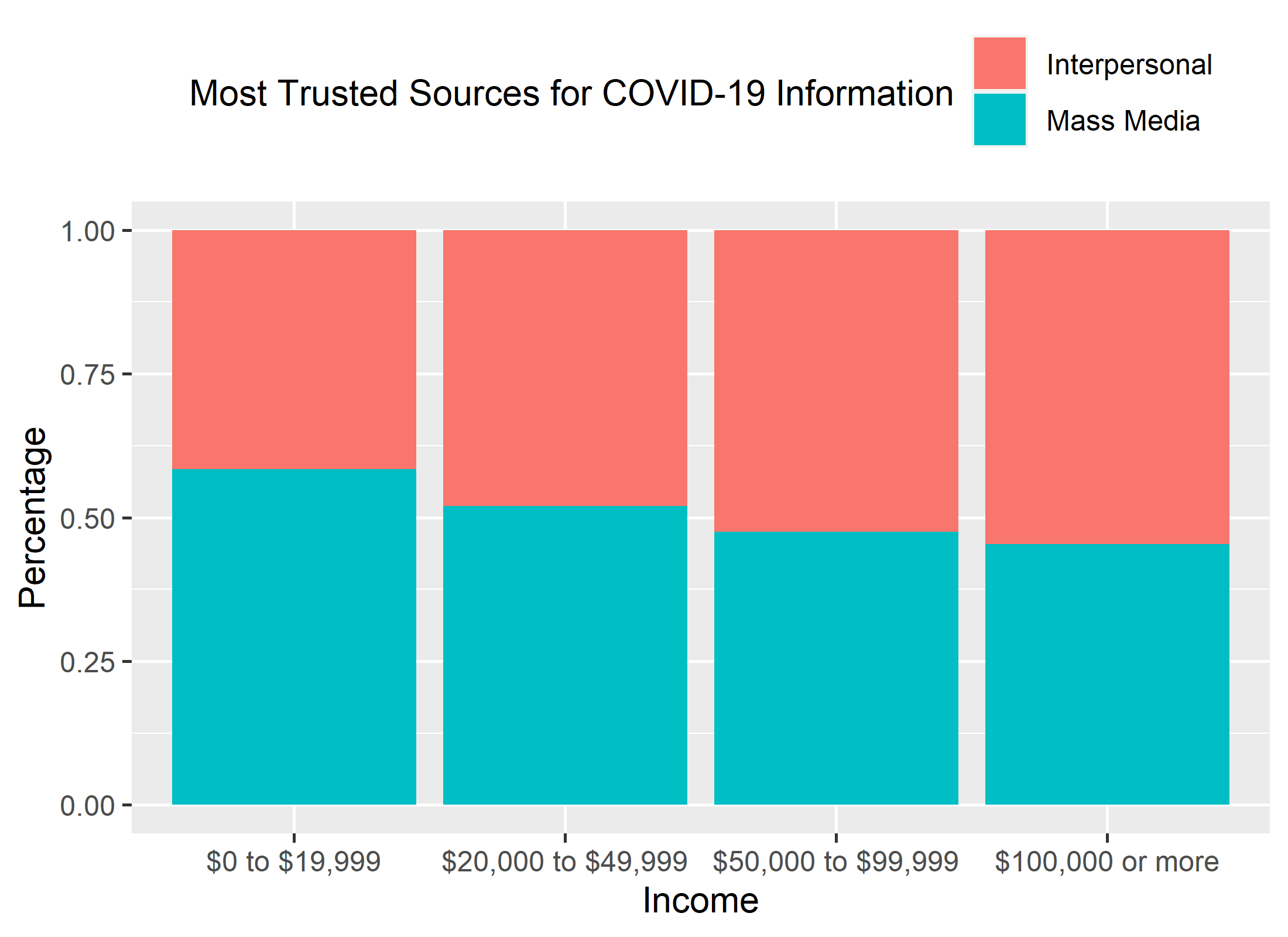

Supplement: Supplementary file 2 — Additional file 2: Appendix 1. Boxplots and bar graphs for predictors of COVID-19 information-seeking. [file 12889_2022_14707_MOESM2_ESM.docx]
